# How well can we separate genetics from the environment?

**DOI:** 10.7554/eLife.64948

**Published:** 2020-12-23

**Authors:** Jennifer Blanc, Jeremy J Berg

**Affiliations:** Human Genetics, University of ChicagoChicagoUnited States

**Keywords:** population stratification, polygenic scores, GWAS, demographic history, None

## Abstract

A simulation study demonstrates a better method for separating genetic effects from environmental effects in genome-wide association studies, but there is still some way to go before this becomes a "solved" problem.

**Related research article** Zaidi AA, Mathieson I. 2020. Demographic history mediates the effect of stratification on polygenic scores. *eLife* **9**:e61548. doi: 10.7554/eLife.61548

A person’s traits – such as their height or risk of disease – result from a complex interplay between the genes they inherit and the environments they experience over their lifetime. To cut through some of this complexity, human geneticists use a tool called a polygenic score, which attempts to predict a person’s traits solely from their genes (Rosenberg et al., 2019).

To build a polygenic score, geneticists first enroll a large number of people in a genome-wide association study (GWAS). For each participant, researchers measure numerous genetic variants across their genome, together with a trait of interest, and use this data to determine the extent to which different variants are associated with the trait. This information makes it possible to take the genome of someone who was not involved in the original GWAS and add up the effects of multiple genetic variants to calculate a polygenic score for that trait (Figure 1A). These scores have been used to predict a person’s risk of developing a disease (Torkamani et al., 2018), to study our evolutionary past (Rosenberg et al., 2019), and to help understand complex social outcomes (Harden and Koellinger, 2020).

**Figure 1.**
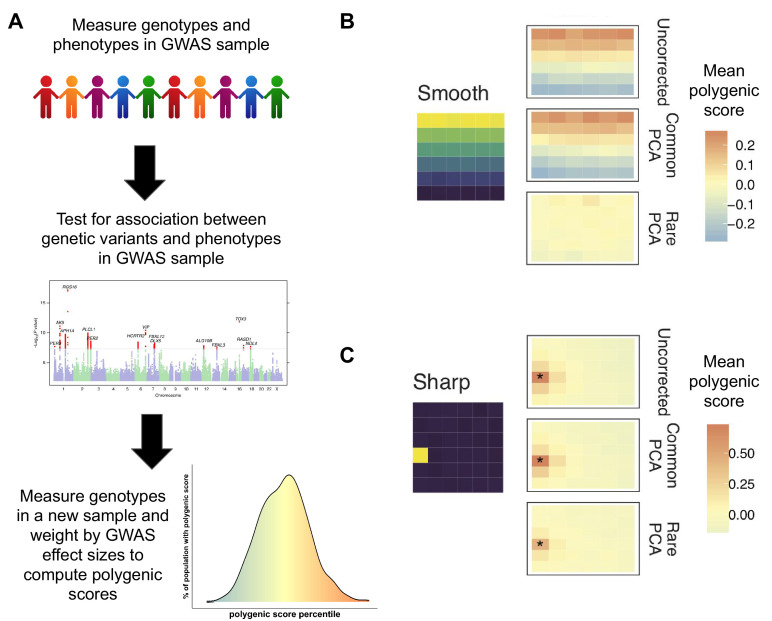
Correcting biases in polygenic scores. (**A**) A genome-wide associate study (GWAS) measures the trait of interest (phenotype) and the genotype of a sample of individuals and uses this data (middle graph) to see which genetic variants (represented by individual dots) are associated with the trait of interest (shown in red). This information is used to compute the polygenic score of individuals not in the original sample. Individuals with a higher polygenic score (orange) are predicted to have a higher trait value (e.g. to be taller or to have a greater risk of disease), while those with a lower polygenic score are predicted to have a lower trait value (bottom graph). (**B**) Mathieson and Zaidi simulated genetic data for a population that separated into subpopulations in the recent past; the environment was simulated as a six-by-six grid (left) in which environmental factors associated with the trait of interest vary smoothly from top to bottom. The uncorrected mean polygenic scores (top right) have a structure that clearly mirrors the structure in the environment. Correcting the scores with the 'common PCA' approach (middle right) does not solve this problem, but correction with the 'rare PCA' approach (bottom right) does. (**C**) However, when differences in the environmental factors were localized to a single square in the grid (shown in yellow), not even the rare PCA model could eliminate the correlation between genetic and environmental effects (indicated by asterix).

Image credit: Panel A – top (Stux, CC0), middle (Figure 1, Hu et al., 2016, CC BY 4.0), bottom (Jennifer Blanc); Panel B (Adapted from Figure 4, Zaidi and Mathieson, 2020).

However, efforts to use polygenic scores face substantial obstacles. All human populations exhibit genetic structure – variation in how genetically similar pairs of individuals are to one another – due to the complex history of geographic separation, population mixtures and migrations that have occurred throughout our evolutionary history. If this genetic structure correlates with patterns of environmental variation, it will cause many genetic variants to be incorrectly associated with a trait. This phenomenon, which is known as population stratification, will introduce biases into polygenic scores and undermine their purpose (which is to separate out the genetic component of trait variation).

To overcome this barrier, researchers would ideally measure the relevant environmental effects in the GWAS sample and include them as statistical controls in their analyses. However, it is difficult – if not impossible – to quantify all environmental effects on a given trait. Existing theory suggests that researchers can use the patterns of genetic variation they have already measured to model the genetic structure of the GWAS sample, and use this as statistical control instead (Song et al., 2015; Wang and Blei, 2019). In essence, because the problem arises from correlations between the environmental effects and patterns of genetic structure, it can be solved by controlling for either of them. The difficulty lies in how to correctly model this genetic structure. Geneticists favor a method called principal components analysis (PCA) (Price et al., 2006), as its simplicity and computational feasibility make it easy to apply to massive GWAS datasets. But the approach has limitations, and population stratification remains an issue in practice (Mathieson and McVean, 2012; Berg et al., 2019; Sohail et al., 2019).

Now, in eLife, Arslan Zaidi and Iain Mathieson from the University of Pennsylvania report which PCA models are the most effective at reducing bias in polygenic scores (Zaidi and Mathieson, 2020). To do this, they simulated the genetic data of a single population which had divided into spatially structured sub-groups within the recent past. They then simulated environmental effects on the trait and tested different PCA models to see how well each model controlled for them.

The results showed that the usual approach, known as ‘common PCA’, leads to polygenic scores that inappropriately mirror the environmental effects. Common PCA models calculate genetic structure by only measuring variants that appear in more than 5% of individuals in the GWAS sample. These common variants are typically ancient in origin, and therefore do not adequately capture the genetic structure of populations which have been spatially subdivided in the recent past. It is this failure to capture the genetic structure that results in biased polygenic scores.

On the other hand, rare variants, which appear in only a handful of individuals, are typically recent in origin and therefore reflect the history of recent subdivisions. Zaidi and Mathieson show that for this reason, PCA models built using patterns of genetic structure in rare variants (‘rare PCA’) eliminate biases from polygenic scores more effectively than the ‘common PCA’ technique (Figure 1B). However, this approach is not a panacea. When the environmental factors associated with the trait were localized to one geographic place (e.g. pollution localized to a particular city), even the rare PCA approach could not separate genetic effects from environmental biases (Figure 1C).

Zaidi and Mathieson also explore a more complicated set of simulations which are meant to more accurately mimic the patterns seen in real GWAS datasets, and find that the results are essentially identical to the simplified scenario described above. In all of their simulations, Zaidi and Mathieson know the ground truth, allowing them to experiment with different approaches designed to target the kind of bias they have simulated. In the real world, the ground truth is not known, so it is difficult to have complete confidence that stratification biases have been properly dealt with. Although a long-studied issue, these findings further demonstrate how separating genetic effects from environmental effects is still not a ‘solved’ problem in genetic studies (Lawson et al., 2020).

Studies that use polygenic scores have exploded in number over the past decade, riding a wave of well-founded optimism that they can open up new, otherwise inaccessible, avenues of research. But care is needed to ensure that this powerful tool is applied appropriately. Ultimately, the possibility for misleading results is an unavoidable risk, especially in research that is restricted to non-experimental settings. Zaidi and Mathieson provide several good recommendations for overcoming this, and suggest that a combination of the rare and common PCA approaches will minimize the amount by which environmental effects confound GWAS data. Moving forward, their results highlight the need for further statistical methods that more effectively deal with the biases introduced by environmental effects, especially for sharply distributed factors. In addition, more sensitive diagnostics are needed to assess how environmental effects impact polygenic scores.
